# Effect of Salt on the Metabolism of ‘*Candidatus* Accumulibacter’ Clade I and II

**DOI:** 10.3389/fmicb.2018.00479

**Published:** 2018-03-16

**Authors:** Zhongwei Wang, Aislinn Dunne, Mark C. M. van Loosdrecht, Pascal E. Saikaly

**Affiliations:** ^1^Biological and Environmental Science and Engineering Division, Water Desalination and Reuse Center, King Abdullah University of Science and Technology, Thuwal, Saudi Arabia; ^2^Department of Biotechnology, Faculty of Applied Sciences, Delft University of Technology, Delft, Netherlands

**Keywords:** water scarcity, saline wastewater, enhanced biological phosphorus removal, phosphate-accumulating organism (PAO), ‘*Candidatus* Accumulibacter phosphatis’ Clade I and II

## Abstract

Saline wastewater is known to affect the performance of phosphate-accumulating organisms (PAOs) in enhanced biological phosphorus removal (EBPR) process. However, studies comparing the effect of salinity on different PAO clades are lacking. In this study, ‘Candidatus Accumulibacter phosphatis’ Clade I and II (hereafter referred to as PAOI and PAOII) were highly enriched (∼90% in relative abundance as determined by quantitative FISH) in the form of granules in two sequencing batch reactors. Anaerobic and aerobic batch experiments were conducted to evaluate the effect of salinity on the kinetics and stoichiometry of PAOI and PAOII. PAOI and PAOII communities showed different priority in using polyphosphate (poly-P) and glycogen to generate ATP in the anaerobic phase when exposed to salt, with PAOI depending more on intracellular poly-P degradation (e.g., the proportion of calculated ATP derived from poly-P increased by 5–6% at 0.256 mol/L NaCl or KCl) while PAOII on glycolysis of intracellularly stored glycogen (e.g., the proportion of calculated ATP derived from glycogen increased by 29–30% at 0.256 mol/L NaCl or KCl). In the aerobic phase, the loss of phosphate uptake capability was more pronounced in PAOII due to the higher energy cost to synthesize their larger glycogen pool compared to PAOI. For both PAOI and PAOII, aerobic conversion rates were more sensitive to salt than anaerobic conversion rates. Potassium (K^+^) and sodium (Na^+^) ions exhibited different effect regardless of the enriched PAO culture, suggesting that the composition of salt is an important factor to consider when studying the effect of salt on EBPR performance.

## Introduction

Application of saline water (seawater or brackish) as secondary quality water for non-potable use such as toilet flushing is a cost-effective and environmentally friendly alternative to mitigate shortage of fresh water in coastline cities and inland areas where brackish ground water is available (WSD, 2009; Leung et al., 2012; Wu et al., 2016). However, this practice will introduce a significant amount of inorganic salt (>1% salinity, considering up to 30% of the fresh water use can be replaced by seawater with the typical salinity of 3.4%) to wastewater treatment plant (Lazarova et al., 2003). Saline wastewaters are also generated from a variety of industrial processes like dairy, seafood processing, vegetable pickling, meat canning and tanneries (Lefebvre and Moletta, 2006). Also, seawater intrusion in the sewer systems can cause elevated salinity of wastewater.

Salt is known to inhibit biological wastewater treatment processes in terms of chemical oxygen demand (COD) removal, nitrification, denitrification and phosphate removal (Hunik et al., 1992, 1993; Kargi, 2002; Moon et al., 2003; Kargi and Uygur, 2005; Moussa et al., 2006; Welles et al., 2014; Corsino et al., 2016). Although many studies reported the effect of salt on organic matter and nitrogen removal, only few studies have focused on the effect of salt on enhanced biological phosphorus removal (EBPR) process. Moreover, the findings of those studies on the effect of salt on EBPR are inconsistent and inconclusive, this is commonly attributed to: (1) differences in operational conditions with respect to temperature, pH and availability of volatile fatty acids (VFAs) (Intrasungkha et al., 1999; Panswad and Anan, 1999; Kargi and Uygur, 2005; Uygur, 2006; Hong et al., 2007; Wu et al., 2008); and (2) interference of other inhibitors such as nitrite (Intrasungkha et al., 1999; Wu et al., 2008; Cui et al., 2009; Bassin et al., 2011). Pronk et al. (2014) reported that the deterioration of phosphorus removal in aerobic granular sludge (AGS) process was mainly due to nitrite accumulation caused by the inhibition of salt on nitrite oxidizing bacteria activities rather than the salt itself when the concentration of salt was below 22 g/L NaCl. Recently, Wang et al. (2017) reported that inhibition of biological phosphorus removal in AGS exposed to 15 g/L NaCl was not due to the accumulation of nitrite (no nitrite accumulation was detected). This discrepancy might be due to the different phosphate accumulating organisms (PAO) clades enriched in the different experiments. In Pronk et al. (2014), all PAOs belonged to ‘*Candidatus* Accumulibacter phosphatis’ Clade I (hereafter PAOI), while in Wang et al. (2017), the granules were enriched by ‘*Candidatus* Accumulibacter phosphatis’ Clade II (hereafter PAOII).

Physiological differences between PAOI and PAOII have been reported in many aspects such as denitrification capability (Flowers et al., 2009; Skennerton et al., 2015), substrate affinities (Slater et al., 2010), temperature preference (Flowers et al., 2013; Tian et al., 2013) and anaerobic metabolic pathway (Welles et al., 2015b). These observations suggest that different PAO clades might have different tolerance or response to salinity. Welles and his colleagues evaluated the short-term effect of salt on enriched PAOII cultures (Welles et al., 2014, 2015a). In the anaerobic phase, PAOII shifted their metabolism from polyphosphate (poly-P)-dependent to glycogen-dependent metabolism with the increase of salinity, and the maximum acetate uptake rate decreased by 71% when the salinity increased to 1% (w/v) (Welles et al., 2014). In the aerobic phase, at 0.18% (w/v) salinity, the corrected phosphate (PO_4_^3-^) uptake rate decreased by 46%; and above 0.35% (w/v) salinity, PO_4_^3-^ was released (Welles et al., 2015a). However, a systematic study comparing the effect of salt on the kinetics and stoichiometry of enriched PAOI and PAOII cultures is lacking.

In addition, all the aforementioned studies only reported the concentration of salt, expressed in units of g/L NaCl, g/L Cl^-^ or w/v %. A large knowledge gap exists regarding the composition of the salt. Potassium ion (K^+^) is one of the abundant cations in seawater (typically 0.38 g/L) (Antonov et al., 2006). More importantly, K^+^ is an important signaling ion to trigger osmo-adaptation processes (Roesser and Müller, 2001). In enhanced biological phosphorus removal process, K^+^ also serves as a counterion of poly-P in PAO activity. It is transported along with phosphate through the membrane during the anaerobic and aerobic phase (Brdjanovic et al., 1998). Thus, it is expected that the effect of KCl on biological phosphorus removal might be different than NaCl.

Therefore, the objectives of this study were (i) to compare the effect of synthetic salt (NaCl or KCl) on the metabolism (stoichiometry and kinetics) of enriched PAOI and PAOII cultures and ii) to evaluate if K^+^ exerts a different impact on P removal compared to Na^+^ at the same mole concentration. This study can contribute to the knowledge on the physiology and biochemistry of different PAO clades in response to Na^+^ and K^+^.

## Materials and Methods

### Enrichment of PAO Cultures in Sequencing Batch Reactors (SBRs)

#### Operation of SBRs

Two identical SBRs, namely SBR1 and SBR2, each with a working volume of 2.75 L, internal diameter of 5.6 cm and a total height of 150 cm, were used in the study. SBR1 was inoculated with 200 mL of aerobic granules (granule mean size, 1.9 mm; circularity, 0.78; settling velocity, 90.2 m/h) collected from a laboratory-scale reactor performing excellent biological phosphorus removal. SBR2 was inoculated with 200 mL of aerobic granules (granule mean size, 1.7 mm; circularity, 0.74; settling velocity, 72.1 m/h) collected from the Garmerwolde Nereda^®^ wastewater treatment plant in The Netherlands. The above two inoculum sources were selected to seed the reactors because they were highly enriched with either PAOI or PAOII. The inoculum used for SBR1 was enriched with PAOI (51%), whereas SBR2 was enriched with PAOII (35%) as determined by quantitative fluorescence *in situ* hybridization (FISH). Glycogen accumulating organism (GAO), a competitor of PAO, was also present in the inoculum (23% in SBR1 and 7% in SBR2).

SBR1 and SBR2 were fed with the same synthetic wastewater. The influent phosphate concentration was 20 mg PO_4_-P/L (0.64 P-mmol/L, 73.1 mg/L K_2_HPO_4_ and 28.6 mg/L KH_2_PO_4_). With the aim to enrich for PAO cultures, a 75%-to-25% mixture of acetate-to-propionate (9.02 C-mmol/L, 300 mg COD/L, 478.4 mg/L NaAc⋅3H_2_O and 64.08 mg/L NaPr) was used as the carbon source (Oehmen et al., 2005). Thus, the influent P/C ratio was 0.07 (P-mol/C-mol). The remaining nutrients were 189.4 mg/L NH_4_Cl, 42.8 mg/L MgSO_4_, 35.0 mg/L KCl, and 1 mL/L trace element solution according to Vishniac and Santer (1957).

The two SBRs were operated in successive cycles of 3 h comprising four phases: 60 min anaerobic feeding from the bottom of the reactors in a plug-flow regimen through the settled bed, 112 min aeration, 3 min settling, and 5 min effluent withdrawal. The air flow was maintained at 4 L/min, pH at 7.0–7.5, and temperature at 22 ± 2°C. The DO concentration was kept at 2.5 ± 0.2 mg/L. The DO concentration was regulated by adding different proportions of compressed air and nitrogen gas, which were controlled by two mass flow controllers (Bronkhorst High-Tech, Netherlands). The gas flow-rate was maintained at 4 L/min during the whole experiment. The exchange ratio of the two SBRs was 0.545, corresponding to a HRT of 5.5 h. Sludge retention time (SRT) was controlled at 20 days. Excess sludge was selectively discharged from the top of the sludge bed to enrich PAO cultures (Winkler et al., 2011).

#### Monitoring the Performance of SBRs

The performance of SBR1 and SBR2 was regularly monitored by measuring mixed liquor suspended solids (MLSS), mixed liquor volatile suspended solids (MLVSS) at a frequency of 10–20 days within one SRT, and the effluent concentrations of acetate (HAc) and propionate (HPr), orthophosphate (PO_4_^3-^-P), ammonium nitrogen (NH_4_^+^-N), nitrite nitrogen (NO_2_^-^-N) and nitrate nitrogen (NO_3_^-^-N) were measured almost on a weekly basis. The SBRs were considered operating at steady state when the concentrations of MLSS and MLVSS were kept stable and complete removal of ammonium and phosphate was achieved. The MLSS and MLVSS were determined according to a modified Standard Methods for the Examination of Water and Wastewaters specifically for AGS (APHA, 2005; Pronk et al., 2014). The ash content was calculated as the ratio of (MLSS-MLVSS)/MLSS. All liquid samples were filtered with 0.45 μm PVDF (polyvinylidine fluoride) filters prior to analysis. NH_4_^+^-N, NO_2_^-^-N, and NO_3_^-^-N were measured by a flow injection analyzer (AutoAnalyzer 3, Germany). The TN was calculated by adding NH_4_^+^-N, NO_2_^-^-N and NO_3_^-^-N. PO_4_^3-^-P was quantified by the ascorbic acid method. Briefly, ammonium molybdate [(NH_4_)_6_Mo_7_O_24_⋅4H_2_O] and antimony potassium tartrate [K(SbO)C_4_H_4_O_6_⋅½H_2_O] react in an acid medium with dilute solutions of orthophosphate to form an intensely colored antimony-phospho-molybdate complex. This complex is reduced to an intensely blue-colored complex by ascorbic acid and the absorbance of the complex is measured at 700 nm wavelength by a UV-VIS spectrophotometer (Shimadzu UV-2550, Japan). VFA (HAc and HPr) concentration was analyzed by a high-performance liquid chromatography (HPLC) (Thermo Scientific, Accela, United States) equipped with a photo-diode array (210 nm) and an ultraviolet detector. The steady-state condition of the reactors was confirmed based on the periodical observations of the above parameters as well as online pH and DO profiles. When both SBRs reached steady-state condition, cycle measurements were carried out as described by Welles et al. (2015b) to compare the behavior of SBR1 and SBR2 enriched with different PAO clades. Poly-β-hydroxyalkanoate (PHA) and glycogen were measured in the cycles according to the method described by Werker et al. (2008). Briefly, 0.5 mL concentrated hydrochloric acid (37% w/v) and 1.5 mL of butyl alcohol were added in a 12-mL glass tube containing 20∼30 mg weighted dried biomass. The tube was capped and incubated at 100°C for 8 h and subsequently cooled down to room temperature. Then, 2.5 mL hexane and 4 mL deionized water were added into the tube to extract the hydrolysis and derivatization products. After vortex mixing (10 s) and subsequent centrifugation (2500 × g for 10 min), one mL of the upper solvent (hexane) phase was transferred to a standard 2-mL GC vial for GC analysis and quantification (Agilent, 7890A, United States). Benzoic acid was used as the method reference standard (RS) for calibration.

#### Identification and Quantification of Microbial Populations

Quantitative FISH was performed 3 times (Day 150, 183, and 202) during the steady-state period to estimate the degree of enrichment of PAO cultures, according to the procedures described in Amman (1995). Fresh granule samples were fixed overnight with 4% (wt/vol) paraformaldehyde. The fixed granules were then crushed and spread on gelatin-coated microscope slides followed by dehydration through sequential immersion for 3 min in 50, 80, and 98% ethanol and air-dried. EUB338mix probe (mixture of probes EUB338, EUB338-II and EUB338-III probes) was used to target the entire bacterial population (Amann et al., 1990; Daims et al., 1999). ‘Candidatus Accumulibacter phosphatis’ was targeted by a PAOmix probe (mixture of probes PAO462, PAO651, and PAO846) (Crocetti et al., 2000), whereas ‘Candidatus Competibacter phosphatis’ (i.e., GAO) was targeted by GAOmix probe (mixture of probes GAOQ431 and GAOQ989) (Crocetti et al., 2002). To distinguish the different PAO clades, PAOI (clade IA and other type I clades) and PAOII (clade IIA, IIC, and IID) were targeted by the probes Acc-I-444 and Acc-II-444 (Flowers et al., 2009), respectively. Hybridization and washing steps on the dehydrated biomass samples were executed under the conditions described by Crocetti et al. (2000, 2002) and Flowers et al. (2009).

To determine the relative abundance of PAO and the fraction of PAO clades (PAOI or PAOII) in the total PAO population (PAOmix), at least 20 images from each sample were taken for the quantification analysis using the Measure/Count tool in Image Plus Pro 6.0 (Media Cybernetics, United States). The relative abundance of PAO was computed by dividing the area of pixels contributed by PAOmix probes to the area of pixels contributed by EUB338mix probes. The fractions of PAO I or PAO II in the total PAO population were computed by dividing the area of pixels contributed by Acc-I-444 or Acc-II-444 probes to the area of pixels contributed by the PAOmix probes.

Quantitative PCR (qPCR) with primers targeting the polyphosphate kinase1 (*ppk1*) gene was used to determine the dominant ‘*Candidatus Accumulibacter phosphatis*’ clades (He et al., 2007; Mao et al., 2015). Total genomic DNA was extracted from the samples using the Power soil DNA isolation Kit (MoBio Laboratories, Inc., Carlsbad, CA, United States) according to the manufacturer’s instructions. The quality (A260/A280) and quantity (A260) of extracted genomic DNA was determined with a Nanodrop^®^ 1000 spectrophotometer (Thermo Fisher Scientific, Waltham, MA, United States). Amplification was performed on a CFX96 Touch^TM^ Real-Time PCR Detection System (Bio Rad Laboratories Inc., Hercules, CA, United States) using iQ SYBR Green Supermix (Bio-Rad, Hercules, CA, United States) with a total reaction volume of 25 μL. All qPCR programs consisted of an initial 3-min denaturation at 95°C, followed by 40 cycles of denaturation at 94°C for 30 s, annealing for 45 s, and extension at 72°C for 30 s. The primer sequences, primer concentrations and annealing temperatures are listed in **Table 1**. For standard clone preparation, the PCR amplicons were first cloned into a TOPO cloning vector (pCR 2.1-Topo vector, Invitrogen, Carlsbad, CA, United States) according to the manufacturer’s protocol. Plasmids from transformed cells were extracted by the PureYield^TM^ Plasmid Miniprep System (Promega, Madison, WI, United States). The accuracy of insert DNA was verified by amplification with gene-specific primers as described in **Table 1**. Copy number per microliter was calculated from the mass concentration and molecular weight of each extracted plasmid DNA. Standard template DNA was 10-fold diluted in series and the C_t_ values for each dilution were plotted against the concentration of each dilution to construct the standard curves. For all unknown samples, 5 ng of community-derived genomic DNA was added as the template. All qPCR assays were performed in triplicate. No-template controls were included in all qPCR runs.

**Table 1:** Primers and qPCR conditions used in this study^a^.

Primer	Sequence (5′ – 3′)	Target	Primer concentration (nM)	T (°C)
Acc-ppk1-763f	GACGAAGAAGCGGTCAAG	Acc-I *ppk1*	500	61
Acc-ppk1-1170r	AACGGTCATCTTGATGGC			
Acc-ppk1-893f	AGTTCAATCTCACCGAGAGC	Acc-IIA *ppk1*	550	61
Acc-ppk1-997r	GGAACTTCAGGTCGTTGC			
Acc-ppk1-870f	GATGACCCAGTTCCTGCTCG	Acc-IIB *ppk1*	400	61
Acc-ppk1-1002r	CGGCACGAACTTCAGATCG			
Acc-ppk1-1123f	GAACAGTCCGCCAACGACC	Acc-IIC *ppk1* excluding	500	63
Acc-ppk1-1376r	ACGATCATCAGCATCTTGGC	OTU NS D3*^b^*		
Acc-ppk1-375f	GGGTATCCGTTTCCTCAAGCG	Acc-IID *ppk1*	400	63
Acc-ppk1-522r	GAGGCTCTTGTTGAGTACACGC			


*^a^The standard used for qPCR was enhanced biological phosphorous removal sludge. ^b^The relative abundance of Accumulibacter in Clade IIC was estimated by qPCR assay using primer sets targeting Accumulibacter IIC excluding OTU NS D3 (He et al., 2007).*

### Anaerobic and Aerobic Batch Tests

Short-term anaerobic and aerobic batch tests were conducted to evaluate the effect of salt on the kinetic and stoichiometric values of enriched PAO cultures cultivated in SBR1 and SBR2 during the steady-state period (Day 181–Day 225). Anaerobic batch tests were conducted to determine the initial specific phosphate release rate (qP_an_), HAc uptake rate (qHAc_an_), glycogen consumption rate (qGly_an_) and PHA formation rate (qPHA_an_) as well as the stoichiometric ratios of total P release/total HAc uptake, total glycogen consumption/total HAc uptake and total PHA formation/total HAc uptake. For each set of anaerobic tests, 4.4 g biomass (in terms of MLSS, ∼80 mL) was taken from each reactor at the end of the cycle (180 min). The granules from each reactor were placed in a 0.2 mm pore size sieve and washed with tap water, and then transferred into a 1-L flask. The flask was filled with tap water up to 250 mL and sparged with nitrogen gas, then 300 mL of deoxygenated influent synthetic medium similar to that fed to the SBRs except the carbon source contained only acetate (300 mg COD/L, 9.38 C-mmol/L) was added into the flask, and a certain amount of salt (NaCl or KCl) according to the salinity tested was introduced into the flask. Nitrogen gas was sparged at 1 L/min for stirring and maintaining anaerobic conditions.

Aerobic batch tests were conducted to determine the initial specific phosphate uptake rate (qP_ae_), glycogen formation rate (qGly_ae_) and PHA consumption rate (qPHA_ae_) as well as the stoichiometric ratios of total P uptake/total PHA consumption and total glycogen formation/total PHA consumption. For each set of aerobic tests, 4.4 g biomass (in terms of MLSS) was collected from each reactor immediately after anaerobic feeding (60 min) and then the biomass from each reactor was subjected to additional anaerobic period in a separate 1-L flask with the addition of 300 mL influent synthetic medium for 2 h to release more phosphate. This step was done to avoid potential poly-P saturation in PAO cells during the following aerobic phosphate uptake process. The granules from each flask were placed in a 0.2 mm pore size sieve and washed with tap water, and then transferred into a 1-L flask, which was filled with tap water up to 250 mL. In the beginning of aerobic batch tests, 300 mL of influent medium containing 200 mg PO_4_^3-^-P/L (6.4 P-mmol/L, 731 mg/L K_2_HPO_4_ and 286 mg/L KH_2_PO_4_) and 5 mg/L allyl-*N*-thiourea (ATU) (to inhibit nitrifiers) was introduced into the flask. There was no carbon source in the medium and the remaining nutrient composition was similar to the medium used in the SBRs. Then a certain amount of salt (NaCl or KCl) according to the salinity tested was introduced into the flask. Compressed air was sparged at 1 L/min for stirring and maintaining the DO close to the saturation level.

Both anaerobic and aerobic batch tests for samples collected from each SBR were performed two times at 5 conditions: no salt addition (control), 10 g/L NaCl (0.171 Na-mol/L), 12.7 g/L KCl (0.171 K-mol/L), 15 g/L NaCl (0.256 Na-mol/L) and 19.1 g/L KCl (0.256 K-mol/L). All percent decreases in the initial specific rates (qP_ae_, qGly_ae_, qPHA_ae_, qP_an_, qHAc_an_, qGly_an_, and qPHA_an_) were compared to the control batch tests without salt addition. The NaCl concentration used in this study was based on the salinity level (∼1%) of municipal wastewater in some coastline cities where seawater (used for toilet flushing) with salinity of 3.4% accounts for 30% of the water used by households (Welles, 2015). The same mole concentration of KCl was used for comparison purposes. The pH was maintained by dosing 0.1 M HCl and 0.1 M NaOH in all batch tests. Each test lasted for 2 h. Samples were collected every 10–20 min for the determination of HAc (anaerobic tests only), PO_4_^3-^-P, PHA and glycogen. To keep a stable SRT, the wastage of excess sludge in SBR1 and SBR2 was adjusted to compensate for the sludge withdrawal due to the batch experiments.

### Calculations

Specific conversion rates were calculated as the slope of the initial linear portion of the profiles of the corresponding chemicals and normalized by the active biomass concentration. The active biomass concentration was determined as MLVSS excluding PHB (Poly-β-hydroxybutyrate), PHV (Poly-β-hydroxyvalerate) and glycogen content (active biomass = MLVSS - PHB – PHV – glycogen), and the concentration was expressed as C-mol/L units by considering the composition of PAO (CH_2.09_O_0.54_N_0.20_P_0.015_) (Smolders et al., 1994a).

Anaerobic ATP production was calculated based on the conversion of polyphosphate (r1) and glycogen (r2) during the anaerobic phase.

$$r1:\underset{\left(polyP\right)}{{HPO}_{3}}+H_{2}O\to ATP+H_{3}{PO}_{4}$$

$$r2:\underset{\left(glycogen\right)}{{CH}_{10/6}}O_{5/6}+\frac{1}{6}H_{2}O\to \frac{2}{3}{CH}_{3/2}O_{1/2}+\frac{1}{3}{CO}_{2}+\frac{1}{2}NADH+\frac{1}{2}ATP$$

Thus, the hydrolysis of 1 P-mol polyphosphate yields 1 mol ATP and 1 mol phosphate; the degradation of 1 C-mol glycogen yields 0.5 mol ATP (Smolders et al., 1994b).

## Results

### Enrichment of PAO Cultures in the SBRs

**FIGURE 1:**
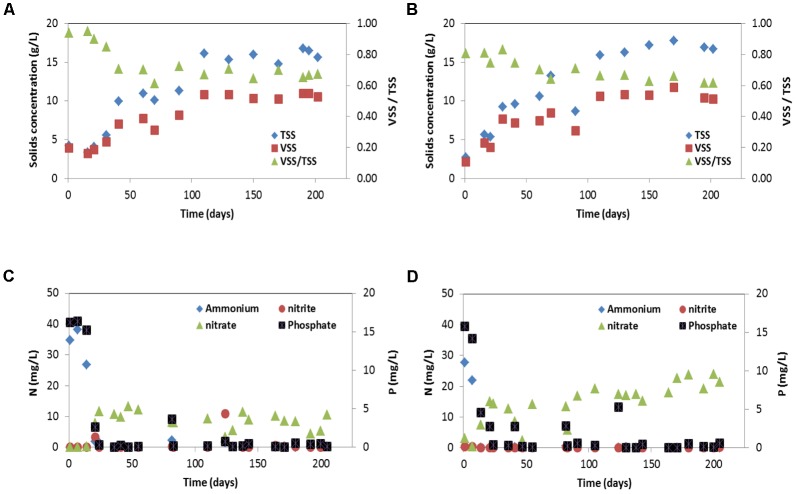
Suspended solids (TSS and VSS) concentrations and effluent concentrations of NH_4_^+^-N, NO_2_^-^-N, NO_3_^-^-N and PO_4_^3-^-P for: **(A,C)** SBR1 and **(B,D)** SBR 2. Suspended solids (TSS and VSS) concentrations were determined at the end of the aerobic phase.

SBR1 and SBR2 were fed with the same synthetic wastewater and operated under the same condition except they were inoculated with a different biomass (51% PAOI and 23% GAO in SBR1 and 35% PAOII and 7% GAO in SBR2). Steady-state conditions were achieved after approximately 130 days of operation. Once the two SBRs reached steady state, the MLVSS content and VSS/TSS ratio were 10.6 ± 0.3 g/L and 67 ± 2% (*n* = 6) in SBR1 and 10.8 ± 0.7 g/L and 63 ± 2% (*n* = 5) in SBR2 during the steady-state period (**Figures 1A,B**). Nitrification and phosphorus removal were gradually established (**Figures 1C,D**), and were accompanied by a decrease of VSS/TSS ratio in both SBRs (**Figures 1A,B**). The removal efficiencies of ammonium and phosphorus reached to more than 98% within 25 (SBR1) to 50 (SBR2) days of operation and nitrite was not accumulated in both SBRs. Effluent nitrate during the steady-state period was constantly lower in SBR1 (8.1 ± 2.5 mg NO_3_^-^-N/L) than in SBR2 (19.8 ± 3.2 mg NO_3_^-^-N/L) (**Figures 1C,D**). At steady state, the granules in both SBRs reached to the same size (2.0 mm in diameter) and had very similar circularity (0.79 in SBR1; 0.77 in SBR2) and settling velocity (91.5 m/h in SBR1; 94.0 m/h in SBR2). The ash content was 33% in SBR1 and 37% in SBR2. The high ash content is characteristic of enriched PAO cultures, indicating high poly-P content (Welles et al., 2014).

**FIGURE 2:**
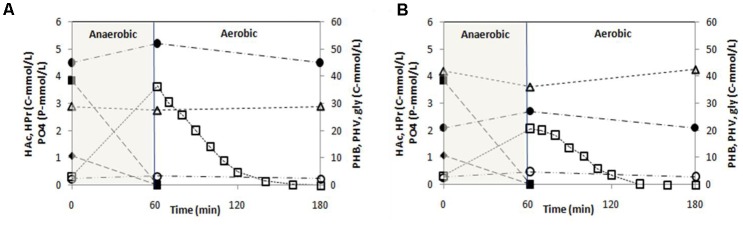
Concentration profiles of polyphosphate (PO4-P), acetate (HAc), propionate (HPr), glycogen (gly), poly-β-hydroxybutyrate (PHB) and poly-β-hydroxyvalerate (PHV) observed during a typical cycle under steady-state conditions in **(A)** SBR1 and **(B)** SBR2. The cycle analyses were conducted on Day 183.

P-release during the anaerobic phase of a typical SBR cycle was 3.30 and 1.73 P-mmol/L in SBR1 and SBR2, respectively (**Figure 2**). All acetate and propionate (4.92 C-mmol/L after dilution) were consumed during the anaerobic phase resulting in a P/HAc ratio of 0.67 P-mol/C-mol and 0.35 P-mol/C-mol in SBR1 and SBR2, respectively. The total glycogen consumption in SBR1 (1.50 C-mmol/L) was lower than in SBR 2 (5.95 C-mmol/L). In the aerobic phase, the specific PO_4_^3-^-P uptake rate was 9.4 P-mmol/(C-mol⋅h) in SBR1 and 4.9 P-mmol/(C-mol⋅h) in SBR2. At the end of the aerobic phase, the active biomass concentration was estimated about 334 C-mol/L in SBR1 and 352 C-mol/L in SBR2.

**FIGURE 3:**
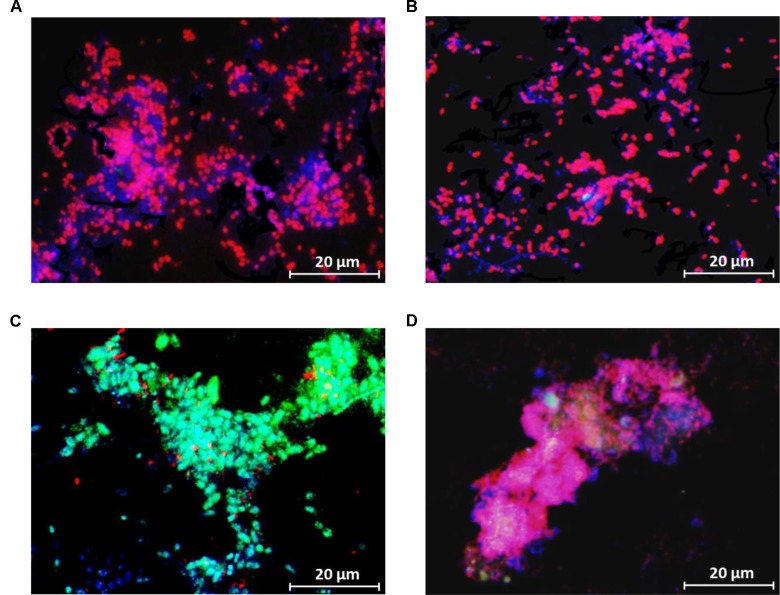
Representative FISH images (the granule samples were taken on Day 183) showing the distribution of bacterial populations in the enriched cultures in **(A,C)** SBR1 and **(B,D)** SBR2. In **(A,B)** blue: EUB mix (Cy5); purple (superposition of blue and red): PAO mix (Cy3); and cyan green (superposition of blue and green): GAO mix (Fluos). In **(C,D)** blue: PAO mix (Cy5), cyan green (superposition of blue and green): PAO clade I (Fluos), and purple (superposition of blue and red): PAO clade II (Cy3).

FISH analysis during the steady-state period demonstrated that ‘*Candidatus* Accumulibacter phosphatis’ were highly enriched in both SBR1 (96 ± 3%) (average of 79 images collected from 3 samples) and SBR2 (95 ± 3%) (average of 70 images collected from 3 samples), as seen from the similar surface area coverage of PAOmix probes and EUB338mix probes in FISH images (**Figures 3A,B**). ‘*Candidatus* Competibacter phosphatis’ was not detected in both reactors. Additional FISH analysis targeting different clades of ‘*Candidatus* Accumulibacter phosphatis’ showed PAOI was the dominant PAO clade (97 ± 5% of total PAO) in SBR1, whereas PAOII was the dominant PAO clade (89 ± 9% of total PAO) in SBR2 and the fraction of PAOI was 9 ± 6% (**Figures 3C,D**). The qPCR results confirmed that PAOI and PAOII were the dominant PAO clades in SBR1 and SBR2, respectively (**Table 2**).

**Table 2:** Relative abundance of specific Accumulibacter clades within the total Accumulibacter lineage^a^.

	I	IIA	IIB	IIC	IID
SBR1^b^	**95.0 ± 3.0%**	∖	0.2 ± 0.1%	4.8 ± 0.9%	∖
SBR2^b^	4.2 ± 1.1%	30.9 ± 1.9%	8.1 ± 2.4%	**56.1 ± 5.6%**	0.7 ± 0.2%


*^*a*^The relative abundance of specific clades within the Accumulibacter lineage was obtained by dividing the *ppk1* copy number of each clade by the sum of the *ppk1* copy numbers from five clades. The numbers in bold indicate the dominant clade among the five clades in each sludge sample. “∖” refers to below the quantification detection limit (10^*3*^ copies/rxn) of the method. ^*b*^The granule samples were taken on Day 183. The ± values were calculated as the standard deviation of triplicate qPCR.*

### Effect of Salt (Batch Tests) on the Anaerobic and Aerobic Kinetic Rates of Enriched PAOI and PAOII Cultures

**FIGURE 4:**
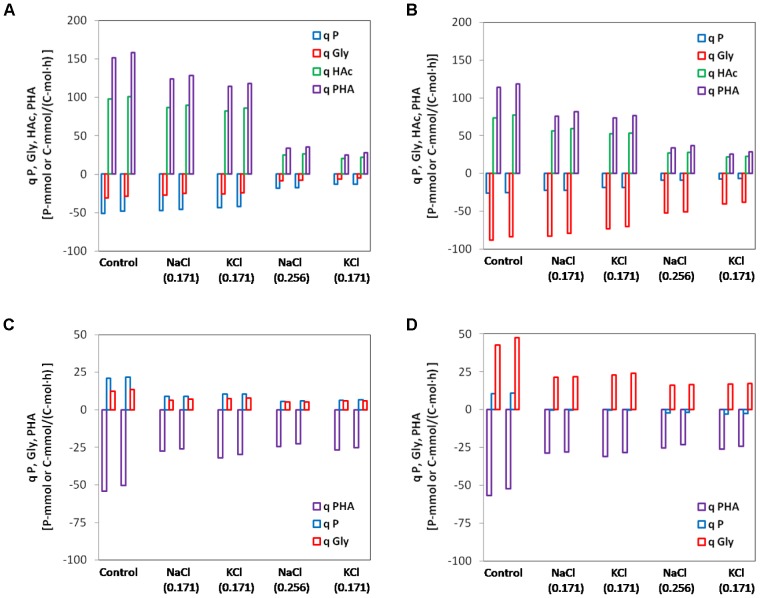
Specific initial conversion rates of polyphosphate (P), glycogen (Gly), acetate (HAc) and poly-β-hydroxyalkanoate (PHA) for the control and different salinity tested. Duplicates at each condition were shown together. **(A)** Anaerobic batch tests with enriched PAOI cultures from SBR1; **(B)** anaerobic batch tests with enriched PAOII cultures from SBR2; **(C)** aerobic batch tests with enriched PAOI cultures from SBR1; **(D)** aerobic batch tests with enriched PAOII cultures from SBR2. The rates of conversions producing ATP were plotted in negative *y*-axis while the rates of conversions consuming ATP were plotted in positive *y*-axis.

All rates were expressed as initial active biomass specific rates based on the profiles of interest (PO_4_-P, HAc (anaerobic tests only), glycogen and PHA) observed in the batch tests. Linear regression was applied to get the slope (concentration/time) of the initial linear portion of the aforementioned profiles. The linear portion lasted at least 30 min and the R-squared values were all above 0.95 except for the PO_4_-P profiles of the biomass from SBR2 at high salinity since the phosphate uptake was almost completely inhibited. The rates of consumption were plotted on negative *y*-axis and rates of production or accumulation are plotted on positive *y*-axis (**Figure 4**).

**FIGURE 5:**
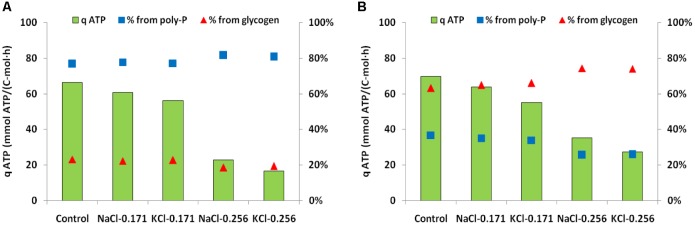
Specific ATP production rates calculated from anaerobic batch tests and the percent contribution to ATP production from poly-P degradation and glycolysis for the control and different salinity tested. **(A)** Anaerobic batch tests with enriched PAOI cultures from SBR1; **(B)** anaerobic batch tests with enriched PAOII cultures from SBR2.

In anaerobic control batch tests, the biomass from SBR1 had a higher qP_an_, qHAc_an_ and qPHA_an_ but lower qGly_an_ (51.1 P-mmol/C-mol, 98.0 C-mmol/C-mol, 151.5 C-mmol/C-mol, 30.8 C-mmol/C-mol) than biomass from SBR2 (25.7 P-mmol/C-mol, 73.5 C-mmol/C-mol, 114 C-mmol/C-mol, and 88.3 C-mmol/C-mol). The addition of 0.171 mol/L NaCl or KCl (10 g/L NaCl or 12.7 g/L KCl) resulted in limited inhibition on the anaerobic kinetic rates of PAOI enriched biomass from SBR1. Compared to the control batch test, the qP_an_, qGly_an_, qHAc_an_ and qPHA_an_ decreased by 7.8, 12.3, 11.2, and 18.0% at salinity of 0.171 mol/L NaCl and 15.1, 16.9, 16.3, and 24.7% at salinity of 0.171 mol/L KCl (**Figure 4A**). When the salinity increased to 0.256 mol/L NaCl or KCl, the inhibition was pronounced as indicated by a sharp decrease (63–83%) in qP_an_, qGly_an_, qHAc_an_ and qPHA_an_ (**Figure 4A**). The overall degree of inhibition on PAOII enriched biomass from SBR2 was similar to SBR1. The qP_an_, qGly_an_, qHAc_an_ and qPHA_an_ decreased by 6–33% at 0.171 mol/L NaCl or KCl, and 40-77% at 0.256 mol/L NaCl or KCl (**Figure 4B**). PAOI enriched biomass had significantly higher qP_an_ and lower qGly_an_ than PAOII enriched biomass (*t*-test, *P* < 0.01). Interestingly, at the same salinity in terms of mole concentration, the specific anaerobic conversion rates measured from NaCl batch tests were constantly higher (*t*-test, *P* < 0.01 for qP_an_, qHAc_an_ and qPHA_an_ in PAOI enriched biomass and qP_an_, qGly_an_, qHAc_an_ in PAOII enriched biomass; *P* < 0.1 for qGly_an_ in PAOI enriched biomass and qPHA_an_ in PAOII enriched biomass) than those measured from the KCl batch tests regardless of the origin of biomass (**Figures 4A,B**). In addition, we calculated the specific ATP production rates according to Smolders et al. (1994b) (**Figure 5**). The enriched biomass from SBR1 (mainly PAO I) and SBR2 (mainly PAO II) had comparable specific ATP production rates at salinity of zero (i.e., control) and 0.171 mol/L NaCl or KCl, but the ATP derived from poly-P degradation was higher in the biomass from SBR1 than SBR2. At salinity of 0.256 mol/L NaCl or KCl, the proportion of ATP derived from poly-P further increased in the biomass from SBR1 (increased by 0.5–1.1% at 0.171 mol/L NaCl or KCl and 5–6% at 0.256 mol/L NaCl or KCl, compared to the control, similarly hereafter) but decreased in the biomass from SBR2 (decreased by 4.6–7.7% at 0.171 mol/L and 29–30% at 0.256 mol/L).

In aerobic control batch tests, the biomass from SBR1 and SBR2 had comparable PHA consumption rates (54.2 C-mmol/C-mol and 56.9 C-mmol/C-mol, respectively). The phosphate uptake rate in the biomass from SBR1 (21.0 P-mmol/C-mol) was 2 times higher than SBR2 (10.6 P-mmol/C-mol), while the glycogen formation rate in the biomass from SBR1 (12.5 C-mmol/C-mol) was 3.4 times lower than SBR2 (42.7 C-mmol/C-mol). Aerobic conversion rates were more sensitive to salt compared with anaerobic conversion rates. For the biomass from SBR1, the qPHA_ae_, qP_ae_, qGly_ae_ dropped by 49, 58, and 49% at salinity of 0.171 mol/L NaCl and 41, 50, and 39% at salinity of 0.171 mol/L KCl (**Figure 4C**). Further inhibition occurred at higher salinity, especially on qP_ae_, which decreased by 74% at 0.256 mol/L NaCl and 70% at 0.256 mol/L KCl (**Figure 4C**). The degree of inhibition on PHA consumption and glycogen formation of the biomass from SBR2 was similar to SBR1. The qPHA_ae_ decrease by 46–56% and qGly_ae_ by 46–62% at all salinity tested (**Figure 4D**). However, the phosphate uptake of biomass from SBR2 was almost negligible at 0.171 mol/L NaCl or KCl, and at 0.256 mol/L NaCl or KCl, phosphate was released (**Figure 4D**). The differences in qP_ae_ and qGly_ae_ between PAOI enriched biomass and PAOII enriched biomass were also significant (*t*-test, *P* < 0.01). In contrast to the phenomenon observed in anaerobic batch tests, at the same salinity in terms of mole concentration, the specific aerobic conversion rates measured from NaCl batch tests were lower than those measured from the KCl batch tests (*t*-test, *P* < 0.05 for qP_ae_, qGly_ae_ and qPHA_an_ in PAOI enriched biomass; *P* < 0.1 for qGly_ae_ in PAOII enriched biomass) regardless of the origin of biomass (**Figures 4C,D**). The difference in the inhibition on aerobic activities between NaCl and KCl was not as evident as those on anaerobic activities. For some aerobic conversions, the difference was smaller than the coverage of standard deviations, probably due to the low aerobic specific conversion rates which resulted in a high relative error.

### Effect of Salt (Batch Tests) on the Anaerobic and Aerobic Stoichiometry of Enriched PAOI and PAOII Cultures

In anaerobic batch tests, the P-release/HAc-uptake ratio (P/HAc) and glycogen-consumption/HAc-uptake ratio (Gly/HAc) of the biomass from SBR1 (mainly PAOI) was stable when the salinity was increased from zero (i.e., control) to 0.171 mol/L NaCl or KCl (**Table 3**), which was in line with the limited inhibition observed by the kinetic rates. At salinity of 0.256 mol/L NaCl or KCl, the P/HAc increased to 0.71–0.75 P-mol/C-mol, whereas Gly/HAc did not vary much with the increase in salinity and remained around 0.33 C-mol/C-mol. The P/HAc of the biomass from SBR2 (mainly PAOII) was stable at all salinity tested, while Gly/HAc increased by approximately 21% at salinity of 0.171 mol/L NaCl or KCl and 70% at sanity of 0.256 mol/L NaCl or KCl (**Table 3**). The PHA formation/HAc uptake ratio (PHA/HAc) slightly decreased by 10–15% at all salinity tested for biomass from both SBR1 and SBR2 (**Table 3**).

**Table 3:** Stoichiometric values of enriched PAOI and PAOII cultures calculated from the batch experiments.

Enriched cultures	Salinity (mol/L)	Anaerobic						Aerobic			
			
		P/HAc (P-mol/C-mol)		Gly/HAc (C-mol/C-mol)		PHA/HAc (C-mol/C-mol)		P/PHA (P-mol/C-mol)		Gly/PHA (C-mol/C-mol)	
						
		Batch 1	Batch 2	Batch 1	Batch 2	Batch 1	Batch 2	Batch 1	Batch 2	Batch 1	Batch 2
PAOI	Control	0.49	0.55	0.29	0.33	1.37	1.51	0.37	0.41	0.21	0.25
	NaCl-0.171	0.52	0.56	0.29	0.33	1.30	1.40	0.31	0.33	0.22	0.25
	KCl-0.171	0.52	0.54	0.29	0.33	1.25	1.35	0.32	0.33	0.23	0.24
	NaCl-0.256	0.72	0.78	0.30	0.36	1.20	1.32	0.20	0.24	0.20	0.22
	KCl-0.256	0.69	0.73	0.26	0.41	1.16	1.46	0.23	0.25	0.21	0.23
PAOII	Control	0.32	0.34	1.15	1.28	1.50	1.62	0.18	0.20	0.66	0.84
	NaCl-0.171	0.37	0.38	1.42	1.56	1.26	1.48	0	0.01	0.72	0.76
	KCl-0.171	0.32	0.34	1.40	1.48	1.35	1.47	0	0.04	0.71	0.77
	NaCl-0.256	0.32	0.34	2.03	2.17	1.24	1.44	-0.12	-0.06	0.62	0.66
	KCl-0.256	0.31	0.33	2.90	2.15	1.05	1.34	-0.07	-0.09	0.62	0.66


In aerobic control batch tests, the P-uptake/PHA consumption ratio (P/PHA) was higher in the biomass from SBR1 than SBR2, whereas the glycogen formation/PHA consumption ratio (Gly/PHA) was lower in the biomass from SBR1 than SBR2 (**Table 3**). The Gly/PHA was largely unaffected by salinity regardless of the origin of biomass. In contrast, the P/PHA decreased with increase in salinity. However, the changes of P/PHA with salinity were different for the biomass from SBR1 and SBR2. The P/PHA in the biomass from SBR1 gradually decreased with the increase in salinity (decreased by approximately 40% at salinity of 0.256 mol/L NaCl or KCl), while the P/PHA in the biomass from SBR2 was close to zero at salinity of 0.171 mol/L NaCl or KCl and was negative at salinity of 0.256 mol/L NaCl or KCl.

## Discussion

### Confirmation of Intrinsic Physiological Differences Between PAOI and PAOII

Despite operating the two SBRs under the same condition for more than 7 months, highly enriched cultures of PAOI and PAOII were cultivated in SBR1 and SBR2, respectively. This suggests that the competitive difference between PAOI and PAOII was small and the type of inoculum having high abundance of PAOI or PAOII was an important parameter for their enrichment. Skennerton et al. (2015) also observed that the inocula were the major factor influencing the Accumulibacter clades enrichment in lab-scale reactors. Similarly, Lopez-Vazquez et al. (2009) simulated PAO/GAO competition indicating the difference in growth rate can be small at some conditions (e.g., pH = 7, temperature = 20°C) that the inoculum becomes important for the outcome of the competition. In addition, both cycle analysis and anaerobic control batch tests showed a relatively high P/HAc and low Gly/HAc for the biomass from SBR1, and a relatively low P/HAc and high Gly/HAc for the biomass from SBR2. Welles et al. (2015b) reported that when poly-P is not stoichiometrically limiting for the anaerobic VFA uptake, PAOI performed the typical PAO metabolism (showing P/HAc of 0.64 P-mol/C-mol and Gly/HAc of 0.29 C-mol/C-mol, pH = 7.0). In typical PAO metabolism, the ATP needed for VFA uptake and PHA formation was mainly derived from poly-P conversion; and the reducing power (NADH) needed for the conversion of VFA into PHA was provided by glycolysis of intracellularly stored glycogen, which also generated some ATP (Mino et al., 1998). In the same study by Welles et al. (2015b) and under similar conditions of no poly-P limitation, PAOII exhibited a mixed PAO-GAO metabolism (showing P/HAc of 0.22 P-mol/C-mol and Gly/HAc of 0.96 C-mol/C-mol, pH = 7.0). In GAO metabolism, ATP and NADH are derived 100% from glycolysis (Zhou et al., 2008). By comparing the anaerobic stoichiometric values in the current study to those obtained by Welles et al. (2015b), it can be confirmed that the biomass from SBR1 and SBR2 used different anaerobic metabolic pathways for VFA uptake. Taken together, the biomass from SBR1 with enriched PAOI culture and SBR2 with enriched PAOII culture exhibited intrinsic physiological differences.

### PAOI and PAOII Respond Differently to Salt

Anaerobic batch tests showed that the proportion of ATP derived from poly-P increased in the biomass from SBR1 but decreased in the biomass from SBR2 at increased salinity. In line with the kinetics results, the P/HAc ratio in the biomass from SBR1 and the Gly/HAc ratio in the biomass from SBR2 increased with the salinity. These results suggest that PAOI relies on poly-P degradation while PAOII on glycolysis for energy production to maintain their anaerobic activities when exposed to high salinity.

Aerobic batch tests showed that the degree of inhibition on PHA consumption and glycogen formation in the biomass from SBR1 and SBR2 were comparable but the inhibition on P uptake was more serious in the biomass from SBR2 (phosphate was even released) than SBR1. The loss of P uptake ability in the biomass from SBR2 at high salinity was probably due to their higher glycogen formation compared with the biomass from SBR1. As the PHA consumption was comparable in the biomass from SBR1 and SBR2, a higher glycogen formation means less PHA can be used for energy production via tricarboxylic acid cycle (TCA) (Smolders et al., 1994a). In addition, the glycogen formation will consume some energy resulting in less energy available for P uptake. In some cases, PAO has to release phosphate to maintain their cell homeostasis as it occurred in this study at salinity of 0.256 mol/L NaCl or KCl. Compared with the biomass from SBR1, during the aerobic phase, the biomass from SBR2 need to convert more PHA and spend more energy to replenish their glycogen stock as more glycogen has been degraded in the previous anaerobic phase (5.95 C-mmol/L in SBR2 vs. 1.50 C-mmol/L in SBR1). On the other hand, the biomass from SBR1 can replenish their relatively small glycogen pool at lower energy cost and still perform P uptake with the remaining energy at the salinity tested.

Moreover, it is worth noting that for the biomass from SBR1, P uptake was more sensitive (70–74% reduction at 0.256 mol/L NaCl or KCl) than glycogen formation (53–58% reduction at 0.256 mol/L NaCl or KCl) with respect to salinity. This preferential inhibition could be explained by the differences in energy demand for each process. According to a stoichiometric model of aerobic metabolism of PAO, the energy demands for poly-P recovery and glycogen recovery were 1.26 molATP/P-mol poly-P and 0.83 mol ATP/C-mol glycogen, respectively (Smolders et al., 1994a). If PAO suffers from any energy limitation, it is logical that glycogen synthesis is less affected than poly-P formation since the former requires less energy.

The degree of inhibition of salt on the anaerobic and aerobic kinetics was different regardless of the origin of biomass. Significant inhibition on all aerobic conversion rates was observed in the biomass from both SBR1 and SBR2. In contrast, the inhibition of salt on anaerobic conversion rates was moderate. This observation that aerobic conversion rates are more sensitive than anaerobic conversion rates to salt is in agreement with the experiment executed on enriched PAOII culture (in the form of flocculent sludge) (Welles et al., 2014, 2015a). In anaerobic phase, PAO can generate energy from both intracellular poly-P degradation and glycolysis of intracellularly stored glycogen. In aerobic phase, energy is usually produced from PHA catabolism and the subsequent oxidative phosphorylation, which is highly sensitive to salinity as indicated by the low half inhibition value (0.2% wt/vol) at which 50% inhibition in O_2_ -uptake rate occurred (Welles et al., 2015a). Currently this seems the best explanation for the aerobic conversion rates being more sensitive to salinity than anaerobic conversion rates. Future studies using metatranscriptomics and proteomics are needed to provide deeper insights on the metabolic shift of different PAO clades in response to salt (He et al., 2010; He and McMahon, 2010; Mao et al., 2014; Barr et al., 2016).

**FIGURE 6:**
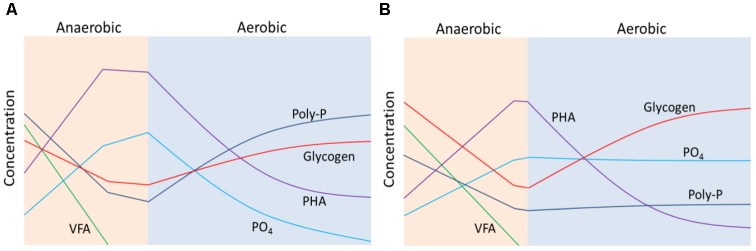
Schematic diagrams showing the change in the concentrations of volatile fatty acids (VFA), phosphate (PO_4_), polyphosphate (poly-P), poly-β-hydroxyalkanoate (PHA) and glycogen during anaerobic-aerobic cycles of enriched **(A)** PAOI and **(B)** PAOII cultures at high salinity.

Based on the above results, schematic diagrams (**Figure 6**) were constructed to depict the conversions of VFA, phosphate, polyphosphate, PHA and glycogen during anaerobic-aerobic cycles of enriched PAOI (**Figure 6A**) and PAOII (**Figure 6B**) cultures at high salinity. In the anaerobic phase, both PAOI and PAOII could uptake sufficient VFA and store it as PHA, and the main source of energy was provided by poly-P and glycogen, respectively. In the aerobic phase, PAOII stored glycogen more quickly than PAOI but was nearly unable to accumulate phosphate. In summary, PAOI is more robust than PAOII in terms of their capability for phosphorus removal at high salinity.

### Comparison of the Effect of Na^+^ and K^+^on PAO Activities

The effect of the same mole concentration of NaCl and KCl on PAO activities was evaluated in this study. The two salts exhibited different effect regardless of the enriched PAO culture. Compared with NaCl, KCl exhibited higher inhibition on anaerobic conversion rates and lower inhibition on aerobic conversion rates. The qP_an_, qGly_an_, qHAc_an_ and qPHA_an_ were 18–27% lower in KCl than NaCl solution, whereas qPHA_ae_, qP_ae_, qGly_ae_ were 11–27% higher in KCl than NaCl. K^+^ is a counterion of PO_4_^3-^ and in the anaerobic phase intracellular K^+^ will be released outside the cells accompanied with PO_4_^3-^. The transportation of K^+^ outside the cell over the cell membrane will cost more energy under high KCl concentration as the transportation of ions against concentration gradient costs energy (Oren, 1999). On the other hand, in the aerobic phase under high extracellular KCl concentration, K^+^ diffuses into the cell down its concentration gradient without energy expenditure during poly-P synthesis (Harold, 1986; Mitchell, 1986).

### Implications and Future Studies

The results of this study revealed that different PAO clades have different tolerance or response to salinity, with PAOI being more tolerant to salt than PAOII. This suggests that for salt water systems inoculation with granular sludge containing PAOI clade is something to consider. Moreover, maintaining adequate functional redundancy might be necessary for enhancing resistance (Saikaly and Oerther, 2011) as different members of PAOI clade might respond differently to salinity. Although there is no solid strategy in the literature to selectively enrich for different PAO clades, it seems based on previous long-term (months to years) lab-scale studies that low COD/P ratio (Welles et al., 2015b) and low temperature (Tian et al., 2013) could select for PAOI culture. Acevedo et al. (2012) observed a significant change in Accumulibacter from Type I to Type II as the poly-P availability decreased in their short-term (18 days) experiment. This suggests that poly-P storage levels can affect the competition between PAOI and PAOII, and could potentially be used as a strategy to enrich for the desired PAO clades. However, unlike laboratory experiments, these strategies might not be practical to implement in full-scale AGS wastewater treatment plants treating saline wastewater. More research is needed to develop practical strategies for enriching PAOI culture in real AGS plants for the treatment of saline wastewater.

The results of the current study were based on short-term (2 h) batch experiments using synthetic saline wastewater containing Na^+^ or K^+^. In general, the tolerance of microorganisms to salt might be increased by long-term adaptation (Bassin et al., 2012). Since K^+^ serves as a signaling molecule to trigger osmo-adaptation processes (Roesser and Müller, 2001), the long-term effect of K^+^ and Na^+^ on PAO might be more different, which need to be studied in the future. In addition, in the case of toilet flushing, the salinity of the wastewater is largely coming from seawater which contains salt-tolerant species, and they might be enriched in the treatment plant after long-term operation affecting carbon and nutrient removal. In addition, seawater contains other ions such as magnesium (Mg^2+^) and calcium (Ca^2+^) that may cause phosphate precipitation (Angela et al., 2011). Also, sulfate (SO_4_^2-^) present in seawater can be reduced by sulfate reducing bacteria (SRB) in wastewater to sulfide (H_2_S/HS^-^), which has an inhibiting effect on many microorganisms including PAO (Comeau et al., 1986; Koster et al., 1986; Rubio-Rincón et al., 2017; Saad et al., 2017). Hence, future studies should be conducted using seawater and evaluated for longer periods.

## Conclusion

The effect of salt on the kinetics and stoichiometry of enriched PAOI and PAOII cultures was compared in this study. The results showed that PAOI and PAOII had intrinsic differences in response to salt inhibition. In anaerobic phase, PAOI uses poly-P as the main energy source to uptake HAc, while PAOII depends on glycolysis of intracellularly stored glycogen to uptake HAc. In aerobic phase, the loss of phosphate uptake capability was more pronounced in PAOII due to the higher energy cost to synthesize their larger glycogen pool compared to PAOI. In general, PAOI was more robust than PAOII in terms of their capability for phosphorus removal at high salinity. Aerobic conversion rates were more sensitive to salt than anaerobic conversion rates. Potassium and sodium ions had different effect on PAOs. Taking the specific conversion rates measured under NaCl conditions as a reference, KCl exhibited stronger inhibition in anaerobic phase than aerobic phase.

## Author Contributions

ZW and PS conceptualized and designed the experiments. ZW and AD performed the experiments. ZW analyzed the data and wrote the manuscript. PS and ML helped in thoughtful discussions and revised the manuscript.

## Conflict of Interest Statement

The authors declare that the research was conducted in the absence of any commercial or financial relationships that could be construed as a potential conflict of interest.
